# Early Local Recurrence as Invasive Micropapillary Carcinoma after Skin-Sparing Mastectomy with Immediate Deep Inferior Epigastric Perforator Flap Reconstruction for Ductal Carcinoma *in situ*

**DOI:** 10.70352/scrj.cr.26-0038

**Published:** 2026-03-27

**Authors:** Takeshi Hashimoto, Minami Masuda, Kurumi Okada, Kazuki Hashimoto, Nao Yamamoto, Seiji Yanai, Sachiko Yuen, Hajime Matsumoto, Takashi Tashiro, Ko Okumura, Kazuhiko Yamagami

**Affiliations:** 1 Department of Breast Surgery and Oncology, Shinko Hospital, Kobe, Hyogo, Japan; 2 Department of Pathology, Shinko Hospital, Kobe, Hyogo, Japan; 3 Department of Plastic Surgery, Shinko Hospital, Kobe, Hyogo, Japan

**Keywords:** ductal carcinoma *in situ*, invasive micropapillary carcinoma, local recurrence, skin-sparing mastectomy, deep inferior epigastric perforator flap reconstruction

## Abstract

**INTRODUCTION:**

Skin-sparing mastectomy with immediate breast reconstruction has been widely adopted to achieve both oncological safety and favorable cosmetic outcomes. Local recurrence after this procedure is generally considered a late event, and early postoperative recurrence at a brief period is uncommon. The mechanisms underlying early local recurrence after skin-sparing mastectomy, particularly in patients with ductal carcinoma *in situ*, remain incompletely understood. We report a rare case of early local recurrence as invasive micropapillary carcinoma following skin-sparing mastectomy with immediate autologous reconstruction for extensive ductal carcinoma *in situ*.

**CASE PRESENTATION:**

A premenopausal woman in her forties was diagnosed with extensive ductal carcinoma *in situ* of the right breast and underwent skin-sparing mastectomy with sentinel lymph node biopsy and immediate breast reconstruction using a deep inferior epigastric perforator flap. The final pathological diagnosis was ductal carcinoma *in situ* with negative surgical margins, and no adjuvant therapy was administered. Ten months after surgery, she noticed a palpable mass in the reconstructed breast, located in the same quadrant as the primary tumor. Imaging studies revealed a well-defined mass, and core needle biopsy demonstrated the invasive micropapillary carcinoma. She was diagnosed with early local recurrence and underwent partial resection of the reconstructed breast. Histopathological examination confirmed invasive micropapillary carcinoma. Retrospective reevaluation of the initial surgical specimen revealed tumor cells with micropapillary architecture adjacent to a biopsy-related disrupted ductal wall, although no definite invasive component had been identified at the time of the initial diagnosis.

**CONCLUSIONS:**

This case illustrates that early local recurrence as invasive carcinoma can occur after skin-sparing mastectomy with immediate reconstruction for extensive ductal carcinoma *in situ*. In addition to residual breast tissue in anatomically vulnerable areas, biopsy-related ductal disruption and aggressive tumor histology were considered potential contributing factors. Careful determination of the resection extent based on tumor location, as well as ensuring the inclusion of biopsy-related tissue changes within the resection field, may be important for improving local control.

## Abbreviations

CNB
: core needle biopsy
DCIS
: ductal carcinoma *in situ*
ddEC
: dose-dense epirubicin and cyclophosphamide
ddPTX
: dose-dense paclitaxel
DIEP
: deep inferior epigastric perforator
ER
: estrogen receptor
FAD
: focal asymmetric density
HBOC
: hereditary breast and ovarian cancer
HER2
: human epidermal growth factor receptor 2
IMPC
: invasive micropapillary carcinoma
MG
: mammography
NME
: non-mass enhancement
PgR
: progesterone receptor
PMRT
: post-mastectomy radiotherapy
SSM
: skin-sparing mastectomy

## INTRODUCTION

In recent years, the concept of oncoplastic breast surgery has been increasingly adopted in the surgical management of breast cancer, aiming to achieve both oncological safety and satisfactory cosmetic outcomes. This approach has become an important treatment strategy for both breast-conserving surgery and mastectomy.^1)^ Among these techniques, SSM involves removal of the whole mammary gland with excision of the nipple–areolar complex while preserving most of the breast skin envelope. When combined with immediate breast reconstruction, SSM is considered to provide acceptable oncological control while maintaining favorable aesthetic outcomes^2)^ Autologous reconstruction using a DIEP flap is one of the most commonly employed reconstructive options, as it allows restoration of a natural breast contour with sufficient volume.

Local recurrence after SSM has been reported to occur predominantly on the skin side of the reconstructed breast and is thought to be associated with residual breast tissue beneath the preserved skin.^3)^ In particular, the upper inner quadrant is recognized as an anatomically vulnerable region where residual glandular tissue is more likely to remain, highlighting the importance of careful determination of resection margins according to tumor location.^3)^ In addition to anatomical factors, from the perspective of oncological safety in oncoplastic surgery, disruption of the ductal wall and tumor cell seeding related to preoperative needle biopsy have been suggested as potential contributors to local recurrence.^4)^

Here, we report a rare case of early local recurrence as invasive micropapillary carcinoma occurring 10 months after SSM with immediate DIEP flap reconstruction for extensive DCIS, with a brief review of the relevant literature.

## CASE PRESENTATION

A 46-year-old premenopausal woman presented to a local clinic in February of year X with a chief complaint of a palpable mass in the right breast. CNB performed at the previous institution raised suspicion of DCIS, and she was referred to our department for further evaluation and treatment. Her past medical history was notable only for uterine fibroids. She had no significant family history of malignancy, no regular medications, and no history of pregnancy or childbirth. She did not meet the established criteria for HBOC screening, and therefore genetic testing for *BRCA1/2* was not performed.

MG, breast US and contrast-enhanced breast MRI were performed at the referring clinic in March of year X prior to CNB. After CNB, US was performed at our institution in June of year X.

**Fig. 1:**
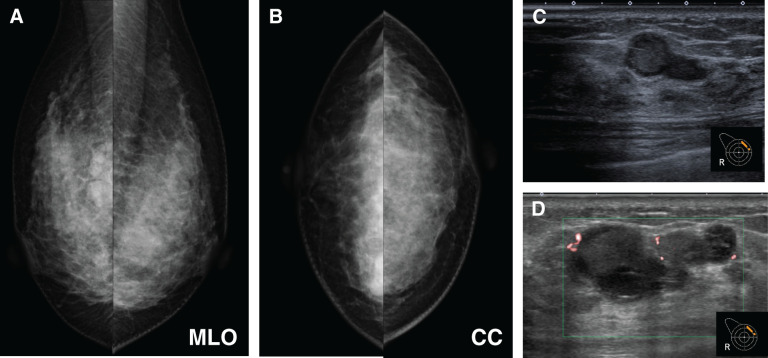
Mammography performed at the referring clinic in March, year X, prior to core needle biopsy, showed focal asymmetric density in the middle to inner region of the right breast (**A**, **B**). US performed at the referring clinic in March, year X, prior to core needle biopsy, demonstrated a 5-mm hypoechoic mass with mixed echogenicity in the upper inner quadrant of the right breast (**C**). US performed at our institution after core needle biopsy demonstrated hematoma-like changes associated with disruption of the cyst wall. The green box indicates the color Doppler sampling box (region of interest), which defines the area evaluated for vascularity using color flow imaging (**D**). CC, cranio-caudal; MLO, medio-lateral oblique

**Fig. 2:**
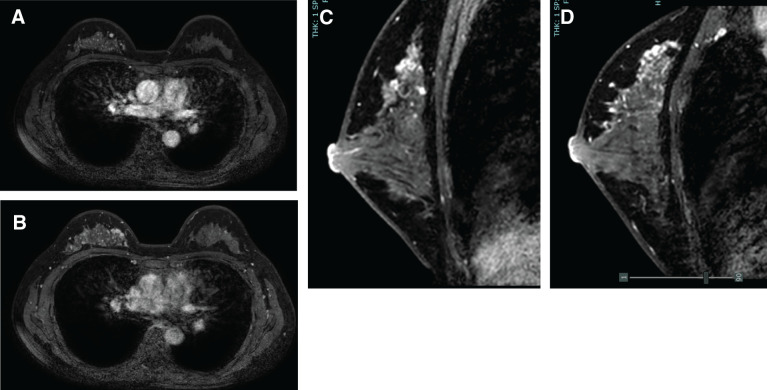
Contrast-enhanced breast MRI demonstrated a 5-mm enhancing nodule, and linear and branching non-mass enhancement with high signal intensity on T1WI from the upper inner quadrant to the upper outer quadrant of the right breast (**A**, **B**). On sagittal images, non-mass enhancement was observed in both the upper inner quadrant to the upper outer quadrant of the right breast (**C**, **D**). T1WI, T1-weighted image

MG demonstrated a FAD in the middle to inner region of the right breast. Breast ultrasonography revealed a 5-mm hypoechoic mass with a heterogeneous internal pattern in the upper inner quadrant of the right breast. After CNB, the lesion exhibited hematoma-like changes associated with disruption of the cyst wall (**Fig. 1**). MRI demonstrated a 5-mm enhancing nodule in the upper quadrant of the right breast, as well as segmental NME extending from the upper inner quadrant to the upper outer quadrant of the right breast, findings consistent with DCIS (**Fig. 2**). The biopsy specimens obtained at the previous institution were reevaluated at our pathological department and confirmed the diagnosis of DCIS.

**Fig. 3:**
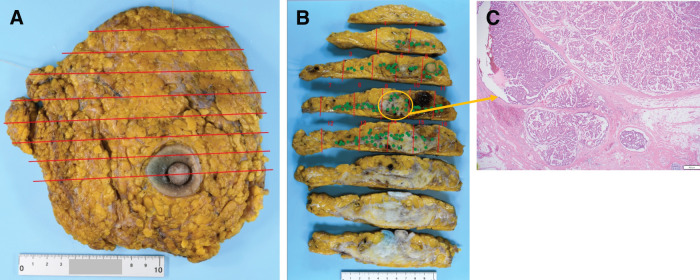
Histopathological examination of the surgical specimen revealed that the tumor extended over an area measuring 12 cm in greatest dimension. The red lines indicate the sectioning planes of the surgical specimen. The green dots indicate the locations where tumor involvement was identified on histopathological examination, mapped onto the corresponding gross specimen sections (**A**, **B**). Tumor cells with moderate nuclear atypia proliferated within dilated ducts, exhibiting papillary to cribriform architectural patterns, and no invasive component was identified (**C**).

Based on these findings, the patient underwent right SSM with sentinel lymph node biopsy, followed by immediate breast reconstruction using a DIEP flap. The skin entry site of CNB was excised by punch biopsy. The final pathological diagnosis was DCIS with negative surgical margins, and no lymph node metastasis was identified. Given these findings, no adjuvant therapy was administered, and the patient was followed up at the referring clinic (**Fig. 3**).

In April of year X+1, 10 months after the initial surgery, the patient noticed a palpable mass measuring approximately 25 mm in the upper inner quadrant of the reconstructed right breast. US demonstrated a well-defined, coarse, hyperechoic mass with a maximum diameter of 23 mm at the same location. No imaging findings suggestive of right axillary lymph node metastasis were observed. CNB of the lesion revealed IMPC, and the patient was referred back to our department for further management.

**Fig. 4:**
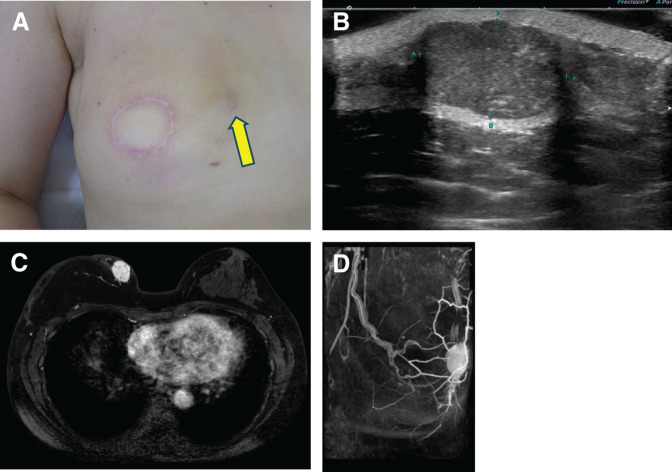
Physical examination revealed a 25-mm elastic hard mass in the upper inner region of the right breast, with thinning of the overlying skin. No palpable axillary lymphadenopathy was detected on the right side. The yellow arrow indicates the location of the tumor (**A**). US demonstrated a well-defined, irregularly shaped mass with internal hyperechogenicity, measuring up to 23-mm in maximum diameter, in the upper inner region of the right breast (**B**). Contrast-enhanced breast MRI demonstrated a 24-mm enhancing mass in the superficial aspect of the upper inner region of the reconstructed right breast (**C**, **D**).

MRI revealed an enhancing mass in the upper inner quadrant of the reconstructed right breast. Additionally, NME was observed on the caudal side of the mass, which was considered to represent tissue changes related to the CNB (**Fig. 4**). Based on these findings, the patient was diagnosed with local recurrence of right breast cancer. In July of year X+1, she underwent partial resection of the reconstructed right breast. In the absence of clinical findings suggestive of lymph node metastasis, additional axillary surgery was considered unnecessary.

**Fig. 5:**
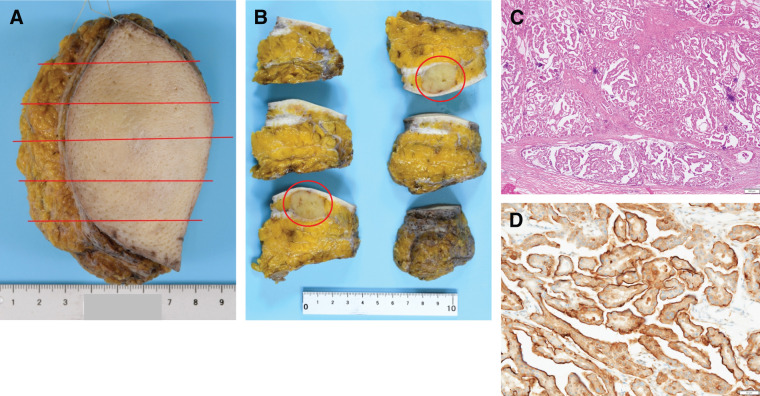
Gross examination revealed a yellowish-white nodule measuring 25-mm in diameter. The red lines indicate the sectioning planes of the surgical specimen. The red circles indicate the gross lesions identified on macroscopic examination of the sectioned specimen (**A**, **B**). On hematoxylin and eosin staining, tumor cells with moderate nuclear atypia exhibited mucin production and a micropapillary architecture, infiltrating the surrounding tissue in an expansile growth pattern (**C**). Immunohistochemical staining for MUC1 demonstrated an inside-out pattern (**D**). MUC1, Mucin-1

Histopathological examination revealed a tumor with a maximum invasive diameter of 25 mm. The tumor exhibited characteristic micropapillary architecture, and immunohistochemical staining for Mucin-1 demonstrated an inside-out pattern, confirming the diagnosis of IMPC (**Fig. 5**). The tumor was ER-positive, PgR-positive, HER2-negative, with Ki-67 labeling index of 25%. No lymph node evaluation was performed at the time of resection.

**Fig. 6:**
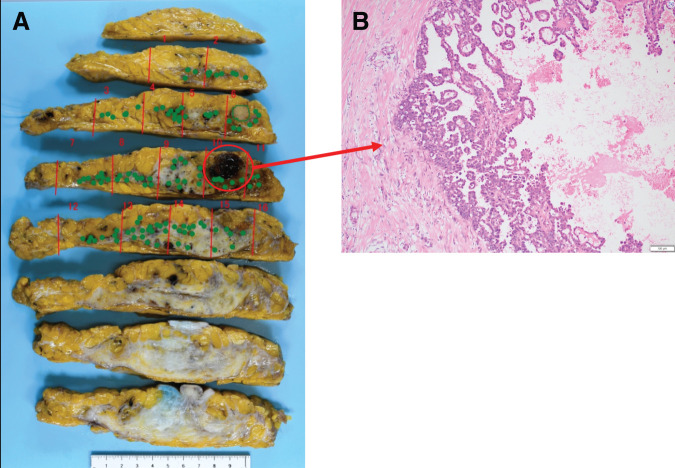
While no definite stromal invasion was evident around the hematoma (hemorrhagic scar), tumor cells showing a micropapillary architecture were identified along the disrupted ductal walls (**A**, **B**). The red lines indicate the sectioning planes of the surgical specimen. The green dots indicate the locations where tumor involvement was identified on histopathological examination, mapped onto the corresponding gross specimen sections.

Furthermore, a retrospective reevaluation of the initial surgical specimen was performed (**Fig. 6**). Tumor cells with micropapillary architecture were observed adjacent to the disrupted ductal wall at the biopsy site; however, no definite invasive component was identified at that time.

Postoperatively, the patient received adjuvant chemotherapy consisting of ddEC, followed by ddPTX. She is currently scheduled to receive adjuvant endocrine therapy, as well as PMRT to the reconstructed breast.

## DISCUSSION

The present case is notable for the development of early local recurrence as IMPC only 10 months after SSM with immediate DIEP flap reconstruction for extensive DCIS. Local recurrence after SSM has generally been reported as a late event, with a median time to recurrence of approximately 4–5 years.^5,6)^ Moreover, most recurrences following SSM are reported to occur on the skin side of the reconstructed breast, where residual glandular tissue may remain beneath the preserved skin flap.^3,7)^ In particular, the upper inner quadrant has been identified as an anatomically vulnerable region with a higher likelihood of residual breast tissue after mastectomy.^3)^ In the present case, both the primary lesion and the recurrent tumor were located in the upper inner quadrant, suggesting that anatomical residual breast tissue may have contributed to local recurrence. However, the unusually short interval to recurrence indicates that factors beyond residual glandular tissue alone may have played a role in the early disease relapse.

One possible contributing factor is the biological aggressiveness of the tumor. IMPC is a distinct histological subtype of breast cancer known to be associated with frequent lymphovascular invasion, high rates of lymph node metastasis, and poor prognosis.^8,9)^ Previous studies have also suggested that DCIS with micropapillary architecture may represent a biologically aggressive entity with an increased risk of progression or recurrence compared with other DCIS subtypes.^10)^ In the present case, although the initial pathological diagnosis was pure DCIS with negative margins, the recurrent lesion demonstrated IMPC, raising the possibility that aggressive biological characteristics were already present at the time of the initial diagnosis. An alternative interpretation is that an occult microinvasive component may have already been present at the time of the initial surgery but remained undetected. The presence of extensive DCIS and biopsy-related hemorrhagic changes could have obscured a small invasive focus, particularly if the invasive component was limited in size and not captured within the examined sections. On retrospective reevaluation of the initial surgical specimen in the present case, no definite invasive component was identified; however, tumor cells with micropapillary architecture were observed adjacent to the disrupted ductal wall at the biopsy site. While these findings do not constitute direct evidence of tumor implantation or early stromal invasion, they raise the possibility that microscopic tumor dissemination may have occurred at a level below the threshold of conventional histopathological detection. Therefore, although both early local recurrence and progression of an initially unrecognized invasive component remain possible, the rapid development of a 25-mm invasive tumor within 10 months may be more consistent with the presence of an occult microinvasive component at the time of the initial surgery.

In addition to tumor biology, the potential impact of preoperative CNB warrants consideration. In this case, disruption of the ductal wall was observed at the site of initial CNB, accompanied by hematoma formation and hemorrhagic scar changes. Tumor cell seeding or implantation related to CNB has been described as a possible phenomenon in which tumor cells migrate into the surrounding stroma through disrupted ductal structures and may subsequently implant along the biopsy tract or within areas of hemorrhage and fibrosis.^4,11)^ Recent case reports have described local recurrence occurring at the biopsy scar or along the needle tract following SSM with immediate reconstruction, suggesting the possibility of biopsy-related tumor displacement or potential seeding, although a direct causal relationship has not been established.^12,13)^ In general, tumors with higher histological grade are considered to have a greater tendency for invasion and metastasis. However, with respect to tumor cell seeding following needle biopsy, high-grade histology is not necessarily the only potential risk factor. It has been reported that factors such as lower nuclear grade and lower Ki-67 index may also be associated with tumor cell seeding, although the clinical significance of these findings remains uncertain.^14)^

When clinical findings such as erythema or induration at the biopsy scar, or imaging findings demonstrating a mass along the needle tract, clearly raise suspicion of tumor seeding, excision of the biopsy skin scar has been suggested.^15)^ Nevertheless, there is currently no definitive evidence supporting routine excision of the biopsy scar in all cases. In patients in whom skin excision is not expected to compromise flap perfusion, individualized consideration of biopsy scar excision may be reasonable.^15)^

Taken together, the early local recurrence observed in this case may have resulted from a combination of multiple factors, including anatomical vulnerability to residual breast tissue in the upper inner quadrant,^3)^ the aggressive biological characteristics of IMPC and DCIS with micropapillary features,^8–10)^ and biopsy-related tissue disruption that could have been associated with tumor cell displacement or implantation.^4,11)^ These factors may have interacted with each other. However, all of these mechanisms remain speculative, and it is difficult to determine the precise cause of recurrence in this individual case.

From a clinical perspective, this case provides important implications for surgical planning and pathological assessment in oncoplastic breast surgery, in case of omission of postoperative radiotherapy as a local control. When performing SSM with immediate reconstruction, it is important, from the perspective of local control, to carefully determine the extent of resection according to tumor location and to ensure that biopsy-related ductal disruption and hematoma sites are included within the resection field. Particularly in cases of extensive DCIS, accurate preoperative assessment of disease extent and appropriate intraoperative adjustment of flap thickness are necessary to achieve complete excision while minimizing the risk of flap necrosis. In SSM, adequate visualization of the medial breast tissue can be technically challenging, and meticulous surgical technique is required.

With regard to adjuvant management, no axillary surgery was performed at the time of recurrence because no clinical or radiological evidence of lymph node involvement was observed. Although high-level evidence specifically addressing PMRT for isolated local recurrence after SSM or NSM with immediate autologous reconstruction is limited, several reports have examined the role of radiotherapy in resected locoregional recurrence and have suggested improved local control in selected patients.^16,17)^ In the present case, considering the early recurrence and the histological characteristics of IMPC, additional PMRT was planned after multidisciplinary discussion and shared decision-making with the patient.

## CONCLUSIONS

In this case, early local recurrence as IMPC was observed only 10 months after SSM with immediate DIEP flap reconstruction for extensive DCIS. In addition to residual breast tissue in the upper inner quadrant, tumor cell implantation at biopsy-related ductal disruption sites may have been associated with tumor cell displacement or potential seeding. When performing SSM with immediate reconstruction, careful determination of the resection extent based on tumor location, as well as ensuring the inclusion of biopsy-related hematoma and ductal disruption sites within the resection field, may be important for improving local control.

## DECLARATIONS

### Availability of data and materials

The datasets supporting the conclusions of this article are included within the article.
